# Editorial: Cytometry in oncology

**DOI:** 10.3389/pore.2026.1612324

**Published:** 2026-01-29

**Authors:** Gábor Barna, József Dudás

**Affiliations:** 1 Department of Pathology and Experimental Cancer Research, Faculty of Medicine, Semmelweis University, Budapest, Hungary; 2 Department of Otorhinolaryngology and Head and Neck Surgery, Innsbruck Medical University, Innsbruck, Austria

**Keywords:** CAR T-cell therapy, hematological malignancy, myelodysplastic syndrome (MDS), myeloma cells, T cell lymphoma

Flow cytometry is a very useful tool not only in research but also in routine oncology. One of its most common areas of application is oncohematological diagnostics. Hematological malignancies were previously diagnosed using microscopy based solely on the morphology of cells: their shape, size, and staining. However, it turned out that B and T cells could not be distinguished just by morphology, and the classification of leukemic cells also proved difficult. Flow cytometry allows us to quickly determine the protein pattern of cells, thereby identifying them. In recent years, flow cytometry has advanced significantly. Devices now contain more lasers and can measure more parameters, allowing us to obtain even more information about examined cells. Of course, every method has advantages and disadvantages. Therefore, using different techniques simultaneously can provide the correct result. This is demonstrated by two articles in our special issue. One of these articles, by Plander et al., discussed how cytometry supports morphology in diagnosing MDS, this precancerous stage. In the article by Szalóki et al., the authors present a rare and noteworthy case of cutaneous T-cell lymphoma, where the combination of morphology and flow cytometry was essential to make an accurate diagnosis.

In recent times, the most dynamically developing field of oncology treatments is the improvement of targeted therapies. One of the most successful targeted therapies is the chimeric antigen receptor (CAR) T cell therapy [1]. It uses the patient’s own, genetically engineered T cells to destroy cancer cells. However, in certain cases, these T cells do not function well or do not persist for long. Particularly interesting were the findings in the paper of Štach et al. in relation to predicting therapy efficacy of CAR-Ts, that the biological characteristics of the tumors, the level of expansion *in vivo*, but not the immunophenotype of produced CAR-T cells, were important parameters. This paper clearly demonstrated a good balance between research and diagnostics, which mutually support each other in development.

The research and diagnostics have always been each other’s driving force. The investigation and understanding of diseases has improved the accuracy and the speed of the diagnostic test, while the identification of diseases and the isolation of cancer cells have created opportunities for new studies. However, it is important to know how the isolation methods change the immunophenotype of the targeted cells. This kind of research was conducted by Czeti et al., in which they used flow cytometry to monitor the immunophenotype of plasma cells isolated by magnetic beads.

It is hoped that the present special issue will encourage a greater number of people to use this diverse and colorful method by demonstrating the diverse oncological uses of cytometry.
